# Effects of Psychiatric Comorbidities on the Prognosis of New-Onset Pediatric Epilepsy: A Retrospective Nationwide Cohort Study

**DOI:** 10.3390/jcm13154500

**Published:** 2024-08-01

**Authors:** Jooyoung Lee, Arum Choi, Sukil Kim

**Affiliations:** 1 Department of Pediatrics, Eunpyeong St. Mary’s Hospital, College of Medicine, The Catholic University of Korea, Seoul 03312, Republic of Korea; jy37jy@naver.com; 2 Department of Preventive Medicine and Public Health, College of Medicine, The Catholic University of Korea, Seoul 06591, Republic of Korea; dyemelody@gmail.com

**Keywords:** psychiatric disorders, children, healthcare utilization rates, retrospective study, status epilepticus, drug-resistant epilepsy

## Abstract

**Background/Objectives:** To determine the impact of psychiatric disorders on epilepsy treatment outcomes and healthcare utilization in children with epilepsy (CWE) based on the presence or timing of the onset of psychiatric disorders. **Methods:** This retrospective controlled study enrolled children (age < 18 years) with newly diagnosed epilepsy into four groups stratified by the presence and timing of the onset of psychiatric disorders (None: no psychiatric disorders; Before: psychiatric disorders only preceding the epilepsy diagnosis; After: new psychiatric disorders diagnosed only after the epilepsy diagnosis; Mixed: different psychiatric disorders diagnosed both before and after epilepsy diagnosis) and compared the intergroup differences in epilepsy treatment outcomes and healthcare utilization. **Results**: Among the CWE (n = 37,678), 13,285 (35.26%) had comorbid psychiatric disorders. The After (n = 7892), Mixed (n = 3105), and Before (n = 2288) groups had significantly longer treatment periods than those in the None group (*p* < 0.001). Compared with the None group, the remaining groups had significantly higher frequencies of outpatient visits, emergency room visits, and admissions and higher rates of status epilepticus and drug-resistant epilepsy (*p* < 0.001, respectively), with higher odds ratios [95% confidence interval] for status epilepticus (2.92 [2.68–3.18]) and drug-resistant epilepsy (3.01 [2.85–3.17]) in the After group. **Conclusions**: Psychiatric comorbidities, diagnosed before and after epilepsy diagnosis, negatively affected the treatment outcomes. CWE without prior psychiatric disorders that were newly diagnosed during epilepsy treatment had the worst outcomes and the highest healthcare utilization rates.

## 1. Introduction

The lifetime prevalence of epilepsy is approximately 1%, and the incidence of epilepsy has a U-shaped distribution by age, with a notable incidence in pediatric patients [1,2]. Children with epilepsy (CWE) frequently present with physical and/or psychiatric disorders [3]. The prevalence of comorbid psychiatric disorders is between 23% and 77%, which is three to seven times higher than that in children without epilepsy [4,5,6,7,8]. In CWE, comorbid psychiatric disorders predominantly include attention-deficit hyperactivity disorder (ADHD), intellectual disability, behavior/emotional disorders, and autism [1,4,6,7]. Several studies have reported that psychiatric disorders increase seizure frequency by lowering the seizure threshold [9,10]. Anti-seizure medication (ASM) or specific types of epilepsy, such as temporal lobe epilepsy, can cause psychiatric disorders [11,12,13], with some studies reporting a bidirectional relationship between epilepsy and psychiatric disorders and the impact of psychiatric comorbidities on CWE. Lower rates of seizure freedom have been reported in CWE with than in those without psychiatric comorbidities [9,14,15]. Comorbid psychiatric conditions negatively affect academic achievement, hinder social adaptation, increase suicide risk, and collectively diminish long-term quality of life [7,16,17,18].

This study aimed to determine the prevalence of psychiatric comorbidities in CWE in South Korea and to evaluate the impact of these disorders on the treatment outcomes of epilepsy. We used a large national CWE sample while considering various factors, such as the incidence of status epilepticus and drug-resistant epilepsy (DRE), and healthcare utilization, including the frequency of admission and emergency room and outpatient visits. We analyzed the differences in prognosis based on the timing of psychiatric comorbidity onset.

## 2. Materials and Methods

### 2.1. Data Source

We used the health insurance claims data of the Health Insurance Review and Assessment Service (HIRA) in South Korea collected from 1 January 2007 to 31 December 2022. Korean National Health Insurance covers 98% of the overall population. The HIRA claims data include diagnosis, surgeries, treatments, and prescriptions [19].

### 2.2. Study Population: Inclusion and Exclusion Criteria

The participants were pediatric patients with epilepsy (age < 18 years) who were newly diagnosed between 1 January 2009 and 31 December 2018. Patients with epilepsy were defined as patients (1) with diagnostic codes corresponding to epilepsy (epilepsy [G40], status epilepticus [G41], and/or seizure [R568]) of the Korean Standard Classification of Diseases (KCD), which is a Korean modification of the International Statistical Classification of Disease and Related Health Problems 10th Revision; (2) who claimed insurance including epilepsy-related diagnostic codes at least twice more than 30 days apart, including inpatients and outpatients; and (3) who were prescribed at least one ASM for 180 days or more during 1 year from the index date, which is the first day with epilepsy-related diagnosis codes with at least one ASM prescription. The ASMs included carbamazepine, clobazam, clonazepam, ethosuximide, gabapentin, lamotrigine, levetiracetam, oxcarbazepine, phenobarbital, phenytoin/fosphenytoin, pregabalin, primidone, rufinamide, topiramate, valproate/valproic acid, vigabatrin, zonisamide, and lacosamide. Febrile seizures (R560) were not considered in this study. Newly diagnosed patients with epilepsy were defined as those (1) meeting the criteria for patients with epilepsy and (2) without epilepsy-related diagnostic codes and ASM prescriptions during the 2-year lookback period from the index date. Children under 2 years of age were included in the study if they did not have an epilepsy-related diagnosis code and an ASM prescription between birth and the index date.

### 2.3. Definition of Psychiatric Disorder

The working definitions of patients with psychiatric disorders were those who used healthcare services more than twice between 1 January 2007 and 31 December 2021, with the same psychiatric diagnoses in the KCD: schizophrenia spectrum disorder and other psychotic disorders (F20–F29), bipolar disorder (F30 and F31), depressive disorder (F32 and F33), anxiety disorder (F40 and F41), obsessive–compulsive disorder (F42), post-traumatic stress disorder (F43), sleep disorder (F51), intellectual disability (F70–F79), communication disorder (F80), specific learning disorder (F81), autism spectrum disorder (F84), attention-deficit hyperactivity disorder (F90), oppositional defiant disorder/conduct disorder (F91 and F92), and tic disorder (F95).

New psychiatric disorders were defined as (1) meeting the criteria for psychiatric disorders and (2) psychiatric disorders diagnosed in patients without previous psychiatric disorders during the 2-year lookback period. For children under the age of 2 years, observation began at birth to determine whether they had been seen more than twice for the same psychiatric condition. The date of diagnosis of psychiatric disorders in patients judged to have a psychiatric disorder was the date when the diagnosis code was first entered. 

Participants were classified into four groups according to the date of the diagnosis of psychiatric disorders compared to the index date as follows: None: no psychiatric disorders; Before: psychiatric disorders only preceding the epilepsy diagnosis; After: psychiatric disorders only after the epilepsy diagnosis; and Mixed: different psychiatric disorders diagnosed both before and after the epilepsy diagnosis. Psychiatric disorders diagnosed after the index date were limited to those diagnosed during the epilepsy treatment period, whereas cases of psychiatric disorders diagnosed after epilepsy treatment had ended were not considered.

### 2.4. Variables

The treatment duration of epilepsy was defined as the period from the index date to the last date of ASM administration. A patient was considered to be on continuous medication if an alternative medication was prescribed within 30 days of the last administration of the previous prescription. Treatment discontinuation was defined as not taking medication for at least six consecutive months from the date of the last medication administration. We identified the number of patients in each group with a primary or secondary diagnosis of status epilepticus (G41) and the number of outpatient and emergency department visits with epilepsy-related diagnoses (G40, G41, and R560) during the treatment period. DRE was defined as a condition wherein a patient simultaneously received three or more ASMs with distinct components in a single prescription or that included diagnosis codes related to intractable epilepsy.

### 2.5. Study Outcome

This study aimed to determine the proportion of CWE in South Korea with comorbid psychiatric disorders and to evaluate the impact of these disorders on epilepsy treatment by comparing the treatment duration of epilepsy, incidence of status epilepticus and DRE, and number of outpatient and emergency department visits according to the presence and timing of the onset of psychiatric disorders.

### 2.6. Statistical Analysis

We used Pearson’s chi-square test to compare the proportions of categorical variables. Owing to the non-normal distribution of the data, we used the Kruskal–Wallis rank sum test to compare the means across the four groups (i.e., None, Before, After, and Mixed). We used Kaplan–Meier and Cox regression analyses, adjusted for age and sex, to examine intergroup differences in the treatment duration for epilepsy. To evaluate the impact of psychiatric disorders on the treatment for epilepsy, relative to the index date of the epilepsy diagnosis, we performed logistic regression analyses for binary outcomes (e.g., presence of status epilepticus, drug-resistant epilepsy) and Poisson regression analyses for the frequency (e.g., number of outpatient visits, ER visits, admissions). We used an alpha level of 0.05 for all statistical tests. Exact *p*-values are reported, and *p*-values less than 0.001 are reported as *p* < 0.001. SAS Enterprise Guide 9.4.2^®^ (SAS Institute Inc., Cary, NC, USA) and R (version 3.5.1; R Foundation for Statistical Computing, Vienna, Austria) were used for the statistical analysis. 

## 3. Results

### 3.1. Participants with Epilepsy and Psychiatric Disorder

**Figure 1:**
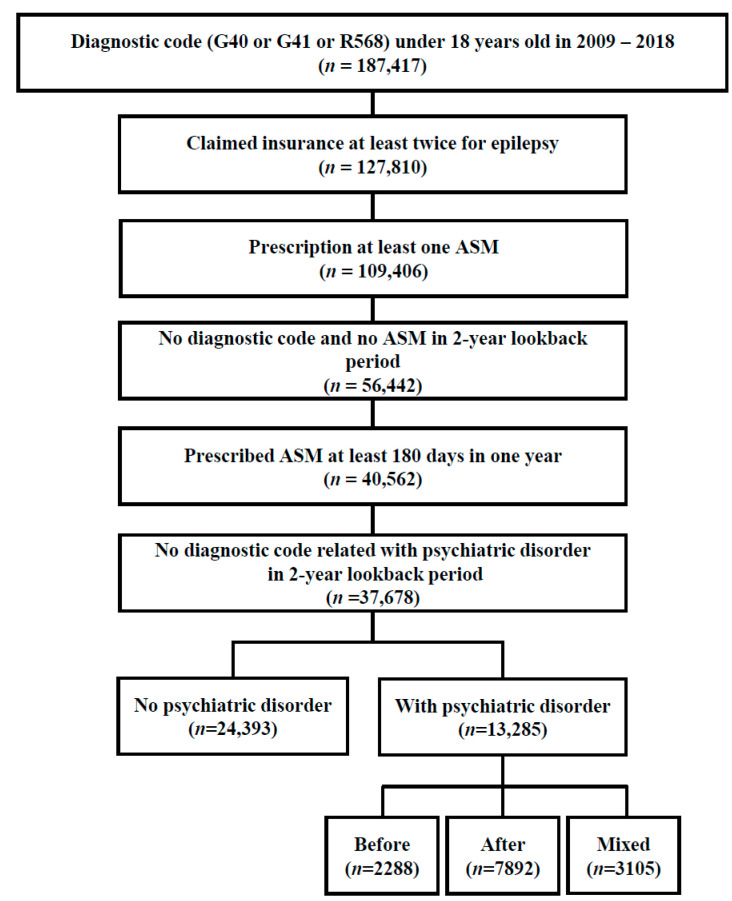
Flowchart depicting the enrollment of the study population. ASM, anti-seizure medication.

From 1 January 2009 to 31 December 2018, 40,562 patients aged <18 years were newly diagnosed with epilepsy and treated with ASM, and 37,678 of these patients remained after applying a 2-year lookback period for psychiatric disorders (Figure 1). A total of 13,285 patients (37.73%) had one or more psychiatric disorders, and the After group had the highest proportion of patients among the groups with psychiatric disorders (59.41%). The total number of patients diagnosed with one or more psychiatric disorders after the epilepsy index date was 10,997, representing approximately a quarter of all CWE. The distribution and type of psychiatric disorders that occurred before and after the index date are shown in Table 1. Patients diagnosed with a psychiatric disorder before the index date had the highest rates of ADHD, followed by anxiety disorder, followed by depressive disorder, and patients diagnosed with a psychiatric disorder after the index date had the highest rates of anxiety disorder, followed by depressive disorder, followed by bipolar disorder. The proportions of males and females diagnosed with each psychiatric disorder are presented in Supplementary Table S1. Except for post-traumatic stress disorder, the proportion of males was significantly higher than that of females for the psychiatric disorders.

### 3.2. Characteristics of the Four Groups

The characteristics of each group are shown in Table 2. All groups had a higher proportion of males than females. The age group with the highest proportion was 5–13 years (42.45%). There were differences in the distribution of groups according to age. The highest proportion of patients in the None group was <1 year old (73.83%), and the lowest proportion was 13–18 years old (56.83%). Patients aged <1 year comprised the highest proportion of patients in the After group (25.15%). Patients aged 13–18 years comprised the highest proportion of those diagnosed with one or more new psychiatric disorders after being diagnosed with epilepsy (After + Mixed group = 35.65%).

### 3.3. Intergroup Differences in Treatment Outcomes and Healthcare Utilization

**Figure 2:**
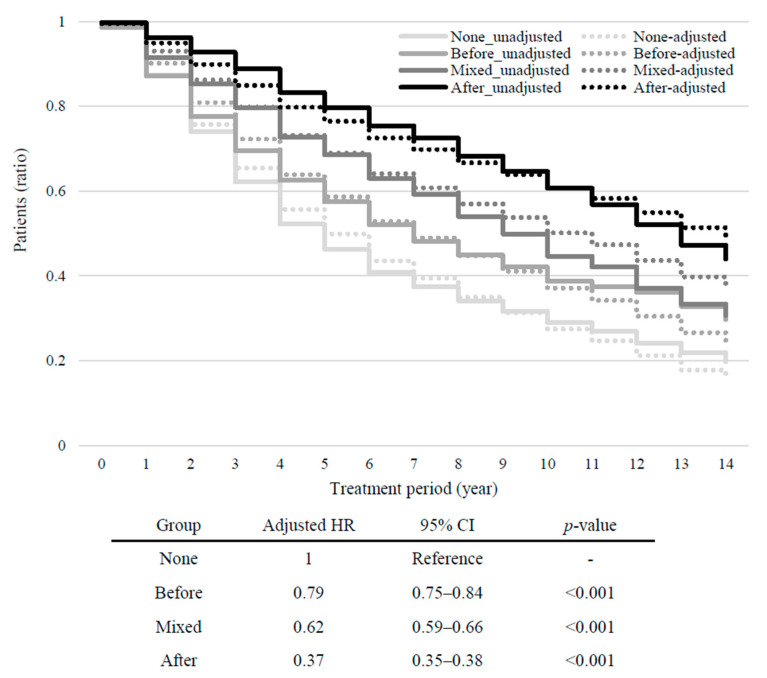
Kaplan–Meier plots showing the proportion of patients who received treatment after the index date. The proportion of patients treated with anti-seizure medication at different time periods from the index date (Time 0) is shown for each group. The solid lines show the unadjusted data. Dotted lines show the data adjusted for age and sex. HR, hazard ratio; CI, confidence interval.

The None group had the shortest treatment duration (5.36 ± 3.59 years, adjusted hazard ratio [HR] = 1, reference), whereas the After group had the longest treatment duration (8.31 ± 3.65 years, adjusted HR = 0.37, 95% confidence interval [CI] = 0.35–0.38, *p* < 0.001), followed by the Mixed group (6.54 ± 3.43, adjusted HR = 0.62 95% CI = 0.59–0.66, *p* < 0.001) and Before group (5.59 ± 3.33, adjusted HR = 0.79, 95% CI = 0.75–0.84, *p* < 0.001) (Figure 2). The number of outpatient visits, emergency room visits, and admissions was approximately double in the After group compared to those in the None group. The total number of medications was the lowest in the None group (2.31 ± 1.83) and the highest in the After group (4.05 ± 2.83), followed by the Mixed (2.88 ± 2.07) and Before (2.46 ± 1.93) groups. Patients diagnosed with status epilepticus and those categorized as DRE had the highest rates in the After group (14.04% and 55.61%, respectively), which were 2.53 and 1.80 times those of the None group.

### 3.4. Regression Analysis on the Healthcare Utilization and Clinical Characteristics for Epilepsy

The associations between the clinical characteristics and healthcare utilization and the presence and timing of the onset of psychiatric disorders are presented in Table 3 and Table 4. The odds ratio (OR) or prevalence ratio (PR) for all clinical characteristics and healthcare utilization was greater than 1.0 in the Before, After, and Mixed groups compared to the None group. For all variables, the strongest association was found in the After group, with a particularly high OR for status epilepticus (PR = 2.92, 95% CI = 2.68–3.18) and DRE (PR = 3.01, 95% CI = 2.85–3.17). Compared to the Mixed group, the After group showed higher correlations for all clinical characteristic variables, with a particularly high PR value for DRE (PR = 1.89, 95% CI = 1.73–2.06), while the Before group had a lower correlation for the total number of medications and DRE (Table 5). However, the Before group did not differ significantly from the Mixed group for status epilepticus.

## 4. Discussion

This large-scale retrospective controlled study using 10-year nationwide data classified psychiatric diseases based on the timing of onset and confirmed the association of psychiatric comorbidities with both treatment response and healthcare utilization in CWE. In this study, we confirmed that CWE co-occurring with psychiatric disorders had worse treatment outcomes for epilepsy than CWE without psychiatric disorders. In particular, CWE who were newly diagnosed with a psychiatric disorder during epilepsy treatment, especially those who had not been diagnosed with a psychiatric disorder before an epilepsy diagnosis, had worse treatment outcomes and more healthcare utilization than the other groups.

Herein, we explain the rationale behind the methodological approach used in our study. To identify patients with epilepsy with sufficient reliability using claims data, we checked not only the number of outpatient visits, including emergency room and admissions with epilepsy-related diagnosis codes, but also medication use [20,21,22,23,24]. Validation studies for all psychiatric conditions in insurance claims data were difficult to find, and many studies have been conducted using disparate methods [4,16,25,26,27,28,29,30]. Using these as guides, to increase the reliability of psychiatric diagnoses, we defined a person with a psychiatric diagnosis if they had at least two visits (admission, outpatient, or emergency room visits) with the same diagnosis code (KCD code: F00-99) as a primary or additional diagnosis. While including medication in the definition of psychiatric diagnosis would have increased the specificity, we did not account for medication status because Korean parents are reluctant to have their children diagnosed with a psychiatric disorder and want to avoid medication as much as possible, which could result in undue under-representation of psychiatric disorders. Given that younger patients with epilepsy are more likely to develop psychiatric disorders during the observation period, we sought to determine the impact of psychiatric disorders on epilepsy treatment by identifying only the psychiatric disorders that occurred during epilepsy treatment after the index date. As DREs are not assigned to the ICD-10 or KCD, additional definitions are needed. According to the International League Against Epilepsy definition, DRE is characterized by a failure to achieve seizure-free status despite the use of two ASMs as a monotherapy or combination therapy [31]. However, insurance claims data do not allow us to determine exactly why the medication was changed or added. Therefore, various methods have been used to define DRE in studies using claims or administrative data [32,33,34,35]. As the most sensitive definition of DRE, we defined DRE as having concurrent prescriptions for three or more ASMs or a diagnosis code related to intractable epilepsy [32].

The proportion of CWE in South Korea with at least one comorbid psychiatric disorder is approximately 40%, which is similar to the proportion in other countries [4,7,36]. In our study, psychiatric disorders were 2.04 times more likely to develop during epilepsy treatment than when epilepsy developed in patients previously diagnosed with a psychiatric disorder. The rates of each psychiatric disorder in CWE vary across studies [4,9,16]. In the present study, the most common psychiatric disorders before and after epilepsy diagnosis were anxiety disorder, depressive disorder, and ADHD (Table 1). ADHD was the most diagnosed psychiatric disorder prior to the diagnosis of epilepsy, whereas anxiety disorders were the most common following the initiation of epilepsy treatment. In our study, we found a higher proportion of males than females for almost all psychiatric disorders. For disorders such as ADHD, autism, learning disorders, and tic disorders, which are known to have a higher prevalence among males in the general population, the proportion of males in our study group exceeded 60%. However, mood disorders, which are typically more common in females in the general population, showed a higher prevalence among males in our study group, which indicates a difference. However, the group with post-traumatic stress disorder had a higher proportion of females, which is consistent with the prevalence in the general population [37,38].

Furthermore, CWE with psychiatric disorders had worse outcomes, such as a longer treatment duration of epilepsy and a higher rate of developing status epilepticus or refractory epilepsy in this study. This finding is consistent with previous studies that have shown lower rates of seizure freedom in patients with comorbid psychiatric conditions [9,14,15]. Comorbid psychiatric disorders may themselves contribute to refractory epilepsy, or conversely, refractory epilepsy may lead to increased medication use, which in turn may increase the incidence of psychiatric disorders [3,10].

This study found that CWE with psychiatric disorders had significantly higher rates of all healthcare utilization than CWE without psychiatric disorders, as reported in previous studies [9,39] and is an expected outcome of increased healthcare utilization associated with a relatively poor prognosis. Similarly, in our study, the highest healthcare utilization was observed in children with psychiatric disorders after epilepsy diagnosis, and these children had the worst outcomes. 

We went beyond merely ascertaining the impact of comorbid psychiatric disorders on epilepsy treatment to evaluating the impact of the timing of the onset of psychiatric disorders. As shown in Table 5, even among CWE with comorbid psychiatric disorders, there were differences in treatment responses based on the timing of their onset. The presence of a psychiatric disorder prior to the diagnosis of epilepsy had less of an association with treatment outcome if no additional psychiatric disorder developed during epilepsy treatment. In contrast, patients who developed a psychiatric disorder during treatment had the worst prognosis, especially if a new psychiatric disorder occurred in patients who did not have a psychiatric disorder before epilepsy treatment. This may be due to differences in the types of psychiatric disorders that occurred before and after epilepsy. However, if these findings were simply because psychiatric disorders had a negative impact on epilepsy treatment, or if they were due to differences in the types of psychiatric disorders that occurred, we would expect to see the worst outcomes in patients who had psychiatric disorders before epilepsy and who developed a new psychiatric disorder after epilepsy (Mixed group). However, our findings contradicted this expectation, with the findings showing the worst treatment prognosis in patients who developed a new psychiatric disorder in the absence of a previous psychiatric disorder (After group). Even if the increased use of ASM in patients with refractory epilepsy and the subsequent development of psychiatric disorders as a side effect could explain the worse treatment outcome in patients who developed psychiatric disorders after epilepsy, explaining the difference between the After and Mixed groups is difficult. Ultimately, we can infer that these results are the result of differences in clinicians being mindful of the development of psychiatric disorders in their patients. Children with pre-existing psychiatric conditions are more likely to develop additional psychiatric conditions. Early detection is associated with timely intervention and better outcomes in psychiatric disorders [26,40]. Therefore, children with pre-existing psychiatric conditions are likely to receive continuous monitoring, which increases the likelihood of early detection and timely treatment of additional psychiatric disorders. In contrast, in patients without pre-existing psychiatric disorders, the lack of ongoing attention to and assessment of psychiatric symptoms is likely to result in a delay in recognizing symptoms; therefore, a longer interval before diagnosis and treatment may have a negative impact on the treatment of epilepsy. Thus, awareness and constant attention to the possibility of psychiatric disorders in CWE are necessary. Furthermore, informing patients and/or caregivers about the possibility of psychiatric disorders, the need for vigilance against the stigma of psychiatric disorders, and the need to report suspicious symptoms early are important to improving the prognosis of epilepsy treatment. Further research is needed on the impact of the timing of onset of psychiatric illness on the prognosis of epilepsy treatment.

This study has several limitations. First, this study was conducted using insurance claims data, which limited the ability to verify each patient’s symptoms and test results. Therefore, it was not possible to reflect differences in treatment outcomes based on the frequency of seizures or the severity of psychiatric symptoms during epilepsy treatment. Second, adherence to ASM may have an impact on the prognosis of epilepsy treatment [41]; however, these data are unavailable in claims data. Therefore, its impact could not be assessed. Third, we were unable to identify epilepsy recurrences for which no diagnostic code was assigned. Therefore, we were unable to determine whether psychiatric disorders were associated with epilepsy recurrence. Finally, this study identified the impact of psychiatric disorders as a whole, and the impact of each psychiatric disorder on epilepsy treatment is difficult to determine. Future studies should explore the effects of individual psychiatric disorders. Furthermore, it is necessary to analyze the difference in the treatment outcomes for epilepsy based on the age at which psychiatric disorders are diagnosed and to determine the specific psychiatric-disorder-stratified treatment outcomes in epilepsy.

## 5. Conclusions

Comorbid psychiatric disorders negatively impact epilepsy treatment and are associated with increased healthcare utilization. A poorer treatment response was observed in children with a newly diagnosed psychiatric disorder after the diagnosis of epilepsy. Among these patients, the worst outcomes and highest healthcare utilization rates were noted in children without a psychiatric disorder before the diagnosis of epilepsy. To reduce the impact of psychiatric disorders on the treatment of epilepsy and to improve the treatment prognosis of epilepsy, the ongoing assessment of psychiatric disorders during epilepsy treatment is necessary, and management to prevent psychiatric disorders in CWE may help improve prognosis.

## Figures and Tables

**Table 1:** The distribution and type of psychiatric conditions occurring before and after the index date.

	Before Index Date	After Index Date	Total
Schizophrenia spectrum disorder and other psychotic disorders (F20 to F29)	451 (1.35)	1483 (4.44)	1934 (5.79)
Bipolar disorder (F30 and F31)	490 (1.47)	2687 (8.05)	3177 (9.51)
Depressive disorder (F32 and F33)	1423 (4.26)	3472 (10.4)	4895 (14.66)
Anxiety disorder (F40 and F41)	1434 (4.29)	4317 (12.93)	5751 (17.22)
Obsessive–compulsive disorder (F42)	116 (0.35)	357 (1.07)	473 (1.42)
Post-traumatic stress disorder (F43)	399 (1.19)	804 (2.41)	1203 (3.60)
Sleep disorder (F51)	191 (0.57)	1108 (3.32)	1299 (3.89)
Intellectual disability (F70 to F79)	1193 (3.57)	2782 (8.33)	3975 (11.9)
Communication disorder (F80)	859 (2.57)	825 (2.47)	1684 (5.04)
Specific learning disorder (F81)	84 (0.25)	171 (0.51)	255 (0.76)
Autism spectrum disorder (F84)	996 (2.98)	1066 (3.19)	2062 (6.17)
Attention-deficit hyperactivity disorder (F90)	1761 (5.27)	2494 (7.47)	4255 (12.74)
Oppositional defiant disorder/conduct disorder (F91 and F92)	352 (1.05)	767 (2.30)	1119 (3.35)
Tic disorder (F95)	574 (1.72)	739 (2.21)	1313 (3.93)

Data are presented as the frequency (proportion). Proportions were calculated from the total number of participants in each category. The index date denotes the date of the first epilepsy-related diagnosis code with at least one ASM prescription.

**Table 2:** Participant characteristics stratified by the presence and timing of diagnosis of psychiatric disorders.

Variables	Total (n = 37,678)	None (n = 24,393)	Before (n = 2288)	After (n = 7892)	Mixed (n = 3105)	*p* *
*Sex*						<0.001
Male	20,569 (54.59)	12,939 (53.04)	1377 (60.18)	4334 (54.92)	1919 (61.80)	-
Age at diagnosis of epilepsy						<0.001
≥0 and <12 months	4470 (11.86)	3300 (73.83)	27 (0.60)	1124 (25.15)	19 (0.43)	-
≥12 months and <5 years	6172 (15.43)	4030 (65.29)	308 (4.99)	1487 (24.09)	347 (5.62)	-
≥5 and <13 years	15,958 (42.35)	10,763 (67.45)	1124 (7.04)	2759 (17.29)	1312 (8.22)	-
≥13 and <18 years	11,078 (29.40)	6300 (56.87)	829 (7.48)	2522 (22.77)	1427 (12.88)	-

Data are presented as the frequency (proportion). The shaded portion of “age at diagnosis of epilepsy” represents the proportion of the four groups at each age and shows horizontal proportions. All other data represent vertical proportions. * *p*-value from the chi-square test.

**Table 3:** Comparison of epilepsy healthcare utilization and clinical characteristics according to the presence and timing of diagnosis of psychiatric disorders.

Variables	None	Before	After	Mixed	p *
Total participants	24,393 (64.74)	2288 (6.07)	7892 (20.95)	3105 (8.24)	
Treatment duration (years)	5.36 ± 3.59	5.59 ± 3.33	8.31 ± 3.65	6.54 ± 3.43	<0.001
No. of outpatient visits	39.97 ± 63.38	44.72 ± 68.14	80.43 ± 127.06	52.09 ± 80.65	<0.001
No. of ER visits	2.68 ± 3.87	2.64 ± 4.32	5.39 ± 8.12	3.41 ± 6.69	<0.001
No. of admissions	3.71 ± 28.10	3.13 ± 18.29	10.76 ± 66.10	3.92 ± 33.69	<0.001
Total no. of medications	2.31 ± 1.83	2.46 ± 1.93	4.05 ± 2.83	2.88 ± 2.07	<0.001
Status epilepticus	1354 (5.55)	154 (6.73)	1108 (14.04)	207 (6.67)	<0.001
Drug-resistant epilepsy	7516 (30.81)	767 (33.52)	4389 (55.61)	1116 (35.94)	<0.001

Data are presented as the frequency (proportion) or mean ± standard deviation. Variable description: “Treatment duration” refers to the period from the index date to the date of the last medication dose. “Total no. of medications” includes the number of ASMs categorized by the ingredient name used at least once during the treatment period. “Status epilepticus” represents the number of patients diagnosed with status epilepticus at least once. “Drug-resistant epilepsy” indicates patients who have been simultaneously prescribed four or more ASMs. ASM, antiseizure medication; ER, emergency room. * *p*-value from the Kruskal–Wallis rank sum test.

**Table 4:** Logistic and Poisson regression analyses of the association of the presence and timing of diagnoses of psychiatric disorders with healthcare utilization and clinical characteristics in pediatric epilepsy (reference: None group).

	None (Reference)	Before	After	Mixed
Number of outpatient visits *	1.00	1.32 (1.29–1.35)	2.83 (2.80–2.85)	1.89 (1.85–1.93)
Number of ER visits *	1.00	1.16 (1.13–1.19)	2.03 (2.00–20.5)	1.59 (1.56–1.63)
Number of admissions *	1.00	1.32 (1.29–1.35)	2.83 (2.80–2.85)	1.89 (1.85–1.93)
Total number of medications *	1.00	1.13 (1.10–1.16)	1.76 (1.74–1.79)	1.35 (1.32–1.38)
Status epilepticus ^+^	1.00	1.69 (1.41–2.01)	2.92 (2.68–3.18)	1.86 (1.59–2.18)
Drug-resistant epilepsy ^+^	1.00	1.34 (1.22–1.47)	3.01 (2.85–3.17)	1.59 (1.47–1.73)

* Poisson’s regression analysis, adjusted for sex and age; odds ratio [95% confidence interval]. **^+^** Logistic regression analysis, adjusted for sex and age; prevalence ratio [95% confidence interval]. ER, emergency room.

**Table 5:** Logistic and Poisson regression analyses of the association of the presence and timing of diagnoses of psychiatric disorders with clinical characteristics in pediatric epilepsy (reference: None group).

	None	Before	After	Mixed
Total number of medications *	0.74 (0.72–0.76)	0.83 (0.81–0.86)	1.30 (1.27–1.34)	1.00 (Reference)
Status epilepticus ^+^	0.54 (0.46–0.63)	0.91 (0.73–1.13)	1.57 (1.34–1.84)	1.00 (Reference)
Drug-resistant epilepsy ^+^	0.63 (0.58–0.68)	0.84 (0.75–0.94)	1.89 (1.73–2.06)	1.00 (Reference)

* Poisson’s regression analysis, adjusted for sex and age; odds ratio [95% confidence interval]. **^+^** Logistic regression analysis, adjusted for sex and age; prevalence ratio [95% confidence interval].

## Data Availability

We obtained health insurance claims data from HIRA for use in our research, and these data are accessible to anyone through approval from the institution. The data provided by HIRA were anonymized and stripped of personally identifiable information before being made available.

## Supplementary Materials

The following supporting information can be downloaded at: https://www.mdpi.com/article/10.3390/jcm13154500/s1, Table S1: Sex-specific Proportions of Psychiatric Disorders in Children with Epilepsy.
