# Supplementary material for: Effects of Psychiatric Comorbidities on the Prognosis of New-Onset Pediatric Epilepsy: A Retrospective Nationwide Cohort Study
Source: J Clin Med. 2024 Aug 1;13(15):4500. doi: 10.3390/jcm13154500 (PMC11312610; doi:10.3390/jcm13154500)
Supplement: Supplementary file 1 [file jcm-13-04500-s001.zip › jcm-3119061-supplementary.pdf]

Supplementary Table S1. Sex-specific Proportions of Psychiatric Disorders in Children with Epilepsy

	**Total**	**Male**	**Female**	***p****
Schizophrenia spectrum disorder and other psychotic disorder (F20 to F29)	1934	1158 (59.88)	776 (40.12)	<0.001
Bipolar disorder (F30 and F31)	3177	1869 (58.83)	1308 (41.17)	<0.001
Depressive disorder (F32 and F33)	4895	2540 (51.89)	2355 (48.11)	<0.001
Anxiety disorder (F40 and F41)	5751	3082 (53.59)	2669 (46.41)	<0.001
Obsessive-compulsive disorder (F42)	473	276 (58.35)	197 (41.65)	<0.001
Post-traumatic stress disorder (F43)	1203	567 (47.13)	636 (52.87)	<0.05
Sleep disorder (F51)	1299	668 (51.42)	631 (40.00)	0.16
Intellectual disability (F70 to F79)	3975	2395 (60.25)	1580 (39.75)	<0.001
Communication disorder (F80)	1684	1106 (65.68)	578 (34.32)	<0.001
Specific learning disorder (F81)	255	153 (60.00)	102 (40.00)	<0.001
Autism spectrum disorder (F84)	2062	1394 (67.60)	668 (32.40)	<0.001
Attention-deficit hyperactivity disorder (F90)	4255	2872 (67.50)	1383 (32.50)	<0.001
Oppositional defiant disorder/conduct disorder (F91 and F92)	1119	748 (66.85)	371 (33.15)	<0.001
Tic disorder (F95)	1313	950 (72.35)	363 (27.65)	<0.001

Data are presented as the frequency (proportion). **p*-values from the chi-square test.
